# Tubercular Mediastinal Lymphadenopathy Presenting as an Isolated Unilateral Vocal Cord Palsy and the “Sail” Sign

**DOI:** 10.7759/cureus.51950

**Published:** 2024-01-09

**Authors:** Alvee Saluja, Shahbaz Anees, Pooja Abbey, L. H. Ghotekar, Rajinder K Dhamija

**Affiliations:** 1 Neurology, Lady Hardinge Medical College, New Delhi, IND; 2 Radio-diagnosis, Lady Hardinge Medical College, New Delhi, IND; 3 Internal Medicine, Lady Hardinge Medical College, New Delhi, IND; 4 Neurology, Institute of Human Behavior and Allied Sciences, New Delhi, IND

**Keywords:** sail sign, vocal cord palsy, mediastinal lymphadenopathy, tuberculosis, recurrent laryngeal nerve

## Abstract

Tuberculosis continues to remain a major public health challenge, especially in low- and middle-income countries. Unilateral vocal cord palsy in adults as the sole manifestation of tubercular mediastinal lymphadenopathy has been rarely reported. A 22-year-old lady presented with a history of hoarseness of voice for the past month. The general physical examination revealed palpable lymph nodes in the left axilla. Axial CT sections at the level of the vocal cords demonstrated dilation of the right laryngeal ventricle and mild anteromedial deviation of the ipsilateral arytenoid cartilage (“sail” sign) suggestive of a right vocal cord palsy. Contrast-enhanced CT chest revealed right paratracheal, right hilar, and subcarinal lymph nodes with areas of central necrosis. She was started on anti-tubercular therapy and her voice completely improved after three months of treatment. The “Sail” sign on axial CT scans is a useful radiological sign for diagnosing unilateral vocal cord palsy. Rarely, compression of the recurrent laryngeal nerve by enlarged mediastinal lymph nodes due to tuberculosis can present with unilateral vocal cord palsy as the sole manifestation in adults.

## Introduction

Tuberculosis (TB) continues to be a significant public health problem, especially in low- and middle-income countries. In 2021, there were approximately 10.6 million individuals afflicted with TB worldwide [1]. India contributes roughly one-fifth to the global TB burden, with an estimated 1.93 million incident cases in 2021 [2].

A recent retrospective study in Spain found that extrapulmonary TB (EPTB) comprised 20.9% of all TB cases [3]. As per the WHO Global TB Report, EPTB comprised 19.5% of TB cases from Southeast Asian regions [1]. Lymph node TB is the most common form of EPTB in India and usually occurs due to the lymphohematogenous spread of *Mycobacterium tuberculosis* [4].

Mediastinal lymphadenopathy due to TB without lung parenchymal involvement commonly occurs in children and is rare in adults [5]. Furthermore, unilateral vocal cord palsy in adults as the sole manifestation of tubercular mediastinal lymphadenopathy has been rarely reported [6,7].

We present a relatively rare case of a young adult female who developed isolated hoarseness of voice and was found to have tubercular mediastinal lymphadenopathy. Moreover, a radiological clue to diagnose unilateral vocal cord palsy is illustrated.

## Case presentation

A 22-year-old lady without comorbidities presented with hoarseness of voice for the past month. She had a history of fever and sore throat for two days before the symptom onset. The patient's initial weight was 47 kilograms and there was a history of weight loss of four kilograms in the last eight months. There was no history of difficulty in swallowing or nasal regurgitation of solids and liquids. There was no loss of smell, vision impairment, double vision, facial numbness or deviation, hearing loss, or reduced tongue movements. She did not complain of weakness or sensory loss in her limbs, and there was no imbalance while walking. She denied a history of cough, shortness of breath, chest pain, night sweats, loss of appetite, malaise and fatigue, lower back ache, abdominal pain, vomiting, altered bowel habits, jaundice, skin rash, joint pains, photosensitivity, recurrent oral/genital ulcers, dry eyes and dry mouth, Raynaud’s phenomenon, and headaches.

She initially sought an opinion from the otorhinolaryngologist due to the persistent hoarseness in her voice. Since her symptoms were preceded by fever and sore throat, the ENT specialist treated her conservatively considering a diagnosis of chronic laryngitis after a viral upper respiratory tract infection. She was given a trial of oral prednisolone for 14 days but reported no significant improvement and was referred for a neurologist's opinion to rule out a central cause for voice hoarseness. On general physical examination, there was no pallor, icterus, cyanosis, clubbing, pedal edema, or thyroid swelling. Palpable matted lymph nodes were noted in the left axilla. Her palatal movements were bilateral and symmetrical, with the uvula centrally placed. The rest of the cranial nerve, pyramidal, extrapyramidal, sensory, and cerebellar system examinations were normal. The complete hemogram revealed a total leukocyte count (TLC) of 6,050 cells/milliliter with a hemoglobin of 11.5 grams/deciliter and a platelet count of 172,000/milliliter. The erythrocyte sedimentation rate (ESR) was 20 millimeters/hour. The liver and kidney function tests were within normal limits. Her thyroid-stimulating hormone (TSH) was 2.50 microIU/milliliter (range - 0.34-5.60). The fasting blood sugar was 101 milligrams/deciliter. Apart from low serum vitamin B12 (144 picograms/milliliter) and 25-hydroxy vitamin D (7.33 nanograms/milliliter) levels, the rest of the routine investigations (including the autoimmune profile) were within normal limits. The Mantoux test was strongly positive (17 mm x 18 mm). Her HIV serology, hepatitis B surface antigen (HBsAg), and anti-hepatitis C antibody were negative.

**Figure 1:**
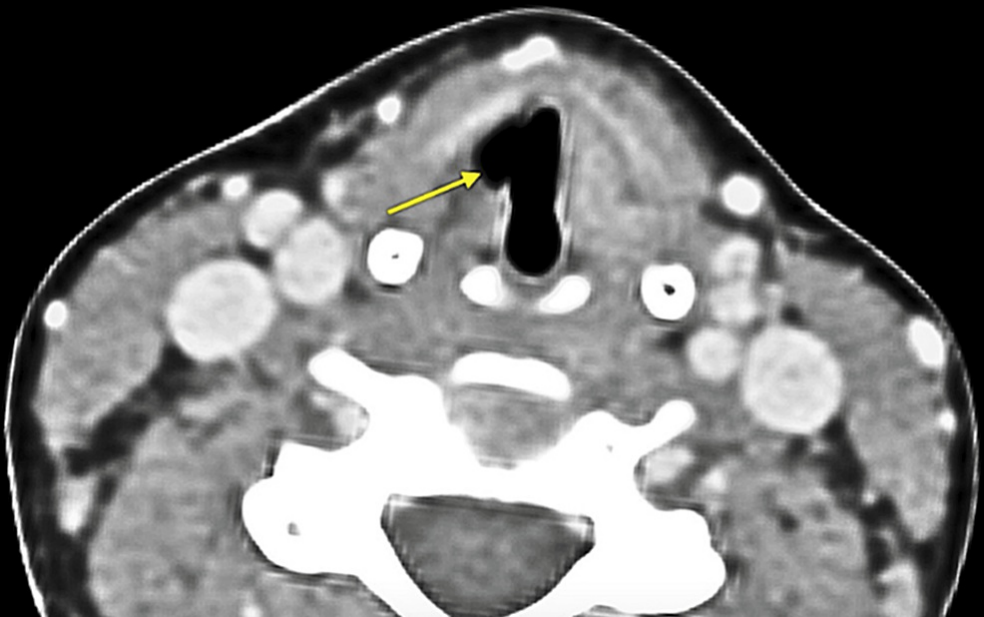
The Sail sign on axial CT sections at the level of the vocal cords Axial CT sections at the level of the vocal cords show dilation of the right laryngeal ventricle (arrow) and mild anteromedial displacement of the right arytenoid cartilage. The dilation of the ipsilateral laryngeal ventricle due to recurrent laryngeal nerve palsy resembles the sail of a ship and is known as the “Sail” sign.

Her magnetic resonance imaging (MRI) of the brain was normal. Axial computed tomography (CT) sections at the level of the vocal cords demonstrated dilation of the right laryngeal ventricle and mild anteromedial deviation of the ipsilateral arytenoid cartilage (“sail” sign) suggestive of a right-sided vocal cord palsy (Figure 1).

A 70-degree endoscopy confirmed the total paralysis of the right vocal cord (Video 1).

**Video 1:** 70-degree endoscopy demonstrating right-sided vocal cord palsy 70-degree endoscopy demonstrates the failure of the right vocal cord to adduct when the patient says “EE” suggesting a right-sided vocal cord palsy.

**Figure 2:**
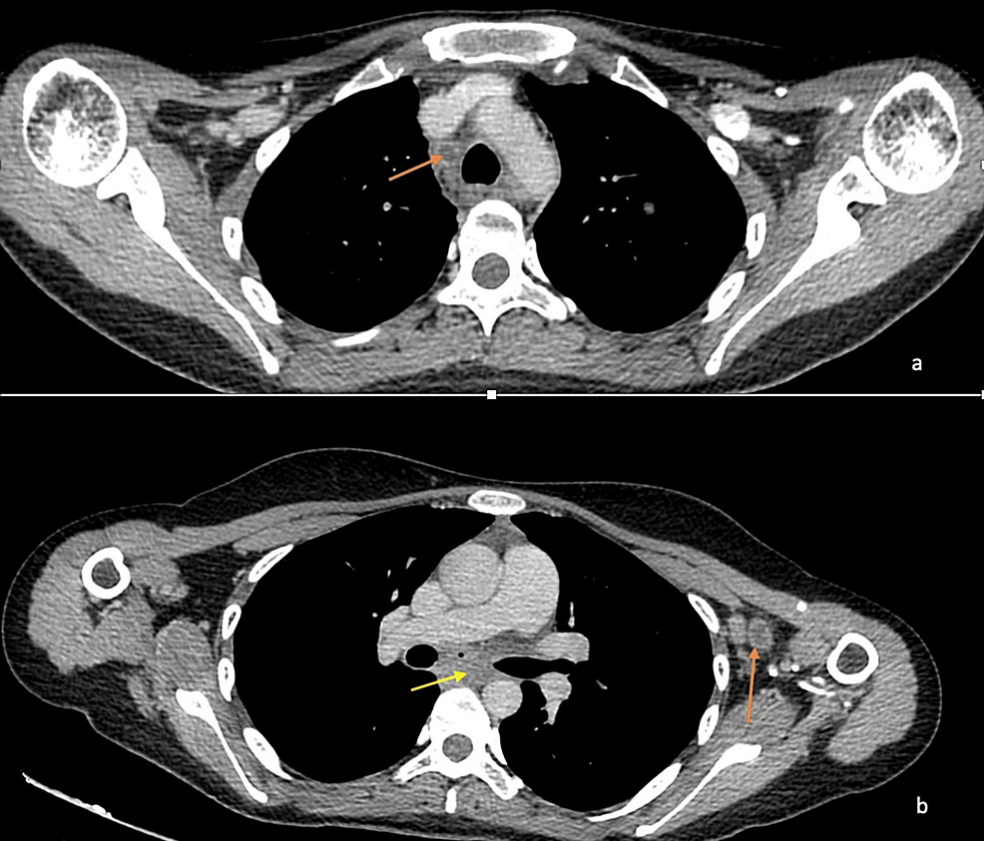
Mediastinal and left axillary lymph nodes due to tuberculosis on the axial sections of the CT chest Axial sections of the CT chest showing (a) a right paratracheal lymph node (orange arrow). A note is made of the central hypodensity within the lymph node suggestive of necrosis and the faint rim of contrast enhancement. (b) A large necrotic lymph node in the left axilla (orange arrow). A subcarinal lymph node is noted as well (yellow arrow).

A contrast-enhanced CT of the chest revealed right paratracheal, right hilar, and subcarinal lymph nodes with areas of central necrosis. Large necrotic left axillary lymph nodes were also noted (Figures 2a, 2b).

Fine needle aspiration cytology from the left axillary lymph node showed the presence of acid-fast bacilli suggestive of TB. The lymph node aspirate was cultured on the Löwenstein-Jensen (LJ) medium and grew *M. tuberculosis *sensitive to rifampicin, after an incubation period of six weeks.

She was started on daily weight-based anti-tubercular therapy (5 mg/kg isoniazid, 10 mg/kg rifampicin, 25 mg/kg pyrazinamide, and 15 mg/kg ethambutol) along with pyridoxine (20 mg once a day) for two months followed by maintenance therapy with isoniazid, rifampicin, and ethambutol at the same dosages for another four months. At three months follow-up, her voice had improved fully.

## Discussion

The etiological differentials of unilateral vocal cord palsy are vast and consist of iatrogenic (post-thyroidectomy, cardio-thoracic surgery, esophagectomy, anterior cervical spine surgery), neoplastic (primary laryngeal, lung, thyroid malignancies or secondary metastatic deposits), traumatic (endotracheal intubation, deceleration injuries), vascular (carotid/aortic dissection, aortic pseudoaneurysm, carotid body tumors, left atrial enlargement, pulmonary artery enlargement), infectious (varicella zoster, TB, histoplasmosis, coccidiomycosis, Lyme’s disease), immune-inflammatory (sarcoidosis, systemic lupus erythematosus, mediastinal fibrosis, silicosis, etc.), demyelination, ischemic (medullary strokes), neuromuscular (myasthenia gravis), and idiopathic causes [8,9].

In the mediastinum, the right recurrent laryngeal nerve (RLN) courses posteriorly under the right subclavian artery to reach the tracheoesophageal groove [10]. Hence, the RLN is especially vulnerable to compression by enlarged hilar and paratracheal mediastinal lymph nodes during its intrathoracic course. The probable route of infection in our case could be due to the lymphohematogenous dissemination of *M. tuberculosis* after it enters the respiratory tract [4]. The proposed mechanisms for RLN palsy in mediastinal TB are compression by adjacent inflamed lymph nodes (the likely mechanism in our case), direct spread of infection from a perforated node that damages the RLN, and a T-cell mediated immune damage involving the RLN due to its proximity to a necrotic lymph node [11,12]. Only a handful of case reports have described isolated unilateral vocal cord palsy as the sole manifestation of enlarged mediastinal lymph nodes due to TB [6,7,11-13]. However, unlike the previous case reports, constitutional symptoms such as persistent low-grade fever, fatigue, rapid weight loss, and loss of appetite were not noted in our case. The ipsilateral dilation of the laryngeal ventricle secondary to thyroarytenoid muscle atrophy on axial CT scans can resemble the sail of a ship (“Sail” sign) and is a useful radiological sign for diagnosing unilateral vocal cord palsy [9].

## Conclusions

We present a rare case of a young adult female who developed subacute onset hoarseness of voice and was found to have mediastinal lymphadenopathy secondary to TB. The presence of the “sail” sign on the CT was a helpful clue in diagnosing unilateral vocal cord palsy, following which further investigations subsequently clinched the diagnosis. This case emphasizes that a high degree of suspicion of primary mediastinal (extrapulmonary) TB must be kept in cases presenting with vocal cord palsy, especially in areas with a high TB burden. Thus, awareness regarding the etiologies of unilateral RLN palsy and a thorough investigative workup can aid in prompt diagnosis and initiation of treatment with good outcomes.
